# Poornima Prabhakaran: balancing development, sustainability and public health

**DOI:** 10.2471/BLT.21.030221

**Published:** 2021-02-01

## Abstract

Poornima Prabhakaran talks to Andréia Azevedo Soares about climate-related health hazards in India, initiatives to address them, and the challenges presented by industrial development.

Q: How did you become interested in the impact of environmental hazards on health?

A: I initially trained as a physician and dreamed of becoming a paediatrician, actually. But then I attended some courses and had the opportunity to work briefly at the Clinical Epidemiology and Biostatistics Division and the Population Health Research Institute at McMaster University in Canada which piqued my interest in epidemiology and set me on a different course. In the end I felt that I could have more of an impact on children’s health by working as an epidemiologist to advance the understanding of drivers of disease, including modifiable environmental factors.

Q: Are children more vulnerable to environmental hazards than other groups?

A: Yes. It is generally accepted that with their greater resting metabolic rates and greater body surface area per unit body weight, children breathe more air per unit body weight than adults do – and consequently more airborne pollutants. They are also exposed to more chemical toxins per kilo of body weight, and being shorter and thus closer to the ground are particularly exposed to ground pollution. Also, when they are very young, they have a tendency to pick things up, touch, taste and put objects in their mouths. As a result, they are more likely to be seriously exposed to a range of toxins, including toxic chemicals and heavy metals such as cadmium, nickel, lead and mercury. Later, as adolescents, they are prone to risk-taking behaviours that can also expose them to environmental hazards.

Q: Which environmental hazards do you focus on at the Centre for Environmental Health?

A: Since its inception in May 2016 the centre has been mainly focused on air pollution, which is a major contributor to morbidity and mortality in India. The challenges we face relate to both outdoor and indoor air pollution. The latter is mainly caused by people using biomass such as wood and dung instead of gas as cooking fuels. According to the *State of global air 2020 *report*,* long-term exposure to outdoor and indoor air pollution contributed to over 1.67 million annual deaths from stroke, heart attack, diabetes, lung cancer, chronic lung diseases and neonatal diseases in India in 2019, while also contributing to over 116 000 infant deaths. We have also established a consortium of exposure scientists and health researchers in India, and here at the centre our team is building a national model for exposure to ambient particulate matter that will help build the evidence base to support future air pollution policy and management in India. We are also working on water, sanitation and hygiene, chemical pollutants, including pesticide use, and climate change.

“In our eagerness to develop quickly, we have not given sufficient consideration to the emissions generated…”

Q: What other environmental challenges does India face?

A: There are several, many of them with a direct link to climate change. Water is a good example. India is a water-stressed country, often subject to droughts. This has direct and indirect impacts on agricultural production, food and nutrition security, besides the economic impacts on farming households. The burden of waterborne diseases is another concern, with outbreaks often relating to acute climate-change-related events. For example, over the past few decades, central India has been subject to a number of catastrophic precipitation events linked to the intense monsoon rains from the southwest which cause rivers to burst their banks. Such events take a tremendous toll on lives and property and often result in overflowing drains and the spread of waterborne pathogens. They can also leave large areas of stagnant water which become breeding grounds for mosquitoes carrying malaria and dengue, among other diseases. It’s also worth noting that climate change is driving a significant redistribution of insect populations in India which is impacting the distribution of disease. For example, we are seeing an increase in the prevalence of vector-borne diseases such as malaria and dengue in north-eastern India. Changing weather patterns are also shortening the time taken by organisms to develop in their intermediate host, boosting the transmission of disease.

Q: Eleven of India’s 15 warmest years on record have occurred since 2004. How are higher temperatures impacting health?

A: In a number of ways. And it is important to note that the country is not just getting hotter overall: the frequency and duration of heatwaves is also increasing. For example, in 2019, the country experienced one of the hottest and longest heatwaves since records began, with the highest temperatures occurring in Churu, Rajasthan where a temperature of 50.8 °C was reported. The heatwave coincided with widespread water shortages and in mid-June, the reservoirs supplying the city of Chennai ran dry. Such heatwaves have multiple health impacts, the most obvious being premature mortality from heatstroke and dehydration, but it also undermines health by impacting agricultural production and increasing food insecurity. In many parts of the country farmers are struggling to maintain crop yields and, in some cases, the nutrient content of the crops produced is decreasing, exacerbating food insecurity issues. Heatwaves and air pollution also contribute to the exacerbation of cardiopulmonary disorders.

Q: How is the national government responding to these challenges?

A: India was among the first countries in the world to draw up a consolidated policy instrument to tackle climate change, with its National Action Plan on Climate Change, which was launched in 2008 under the aegis of the Prime Minister’s Council on Climate Change. The Ministry of Health provides support to the programme through the National Health Mission, and a National Action Plan for Climate Change and Human Health was prepared in 2018. The main aim of the plan is to strengthen health service delivery in response to the adverse impacts of climate change on health. The National Centre for Disease Control, the nodal agency for the National Programme for Climate Change and Human Health (NPCCHH), is working with states and union territories across India to develop state-specific climate action plans. They are supported by Centres of Excellence based in nationally reputed institutions in specific domains ranging from air pollution, water pollution, vector-borne diseases, cardiopulmonary diseases, zoonotic diseases, nutrition, mental health, occupational health, disaster management, vulnerability assessments and green and climate resilient health-care facilities. However, as important as these different initiatives are, like many industrializing countries, India struggles to balance economic growth and sustainability.

“The pandemic […] presents us with an opportunity to think about the kind of country we want to be going forward.”

Q: How is that struggle manifested?

A: The level of emissions is one area. The government has been criticized for prioritizing economic development over emission reduction targets. For my part, I believe that in our eagerness to develop quickly, we have not given sufficient consideration to the emissions generated by different sectors. And I am not just talking about greenhouse gases or air pollution. It is true, for example, of the wastewater generated by our industries as well, including the pharmaceutical industry. India is known as the pharmacy of the world, and Indian manufacturers make active pharmaceutical ingredients and a huge volume of finished pharmaceutical products, all of which is very positive, but it has a significant impact on the environment. The health system itself also impacts the environment, notably in the generation of greenhouse gases during service delivery, resource inefficiency and through the generation of waste – both solid waste and biomedical waste besides a whole range of end-of-lifecycle medical equipment, electronic waste and used packaging.

Q: Can you talk about your work on reducing the environmental impact of the health system?

A: Since 2016, we have been collaborating with the global nongovernmental organization Health Care Without Harm (HCWH) which works to reduce the environmental footprint of health systems and boost health system climate resilience – so-called climate smart health care. It is in working with HCWH that I have come to see the possibilities for pursuing a climate smart health-care agenda in India.

Q: How successful have you been in pursuing that agenda?

A: It has been challenging to get our message heard, but there are signs that the climate and health issue is moving up the political agenda. This is partly because climate change is becoming a mainstream issue, and partly because greening has been an important component in discussions regarding the post-pandemic economic recovery. The NPCCHH prioritizes addressing air pollution and heat-related illnesses and is now working on the creation of green and climate resilient health-care facilities, a project for which we are creating a national development framework. The plan is to use the national framework developed under the NPCCHH to guide the development of State Action Plans that will be implemented across the public health system. However, there is also a vast network of private health-care providers across the country and we will need to sensitize these providers too if we want to make progress on the climate smart health agenda.

Q: What impact has the COVID-19 pandemic had on the climate smart health discussion in India?

A: As in other countries, the pandemic has highlighted the inherent inequities in the health system, the public sector component of which is severely underfunded. I would say there is now an increasing recognition that we need greater investment in public health, including in training health professionals. The pandemic also presents us with an opportunity to think about the kind of country we want to be going forward. While the economic shutdown has been terrible and has caused great hardship to many, it has also reminded us of the price we have paid for rapid industrialization and continuing dependence on coal-fired power generation and industrial activity. We had not seen blue skies in a long time in Delhi, where I live. We saw them during the lockdowns. Similarly, we have never been more aware of the importance of health, health care and of the environment. We need to seize this moment to reconsider our priorities and to plan for the future accordingly.

